# Kinetics Study of Microwave-Assisted Brine Extraction of Lipid from the Microalgae *Nannochloropsis* sp.

**DOI:** 10.3390/molecules25040784

**Published:** 2020-02-12

**Authors:** Nour Zghaibi, Rozita Omar, Siti Mazlina Mustapa Kamal, Dayang Radiah Awang Biak, Razif Harun

**Affiliations:** 1 Department of Chemical and Environmental Engineering, Faculty of Engineering, University Putra Malaysia, Serdang 43400, Selangor, Malaysia; dradiah@upm.edu.my (D.R.A.B.); mh_razif@upm.edu.my (R.H.); 2 Department of Process and Food Engineering, Faculty of Engineering, University Putra Malaysia, Serdang 43400, Selangor, Malaysia; smazlina@upm.edu.my

**Keywords:** microwave-assisted extraction, brine, kinetics, lipid, microalgae, polyunsaturated fatty acids

## Abstract

The kinetics of lipid extraction utilizing microwave-assisted extraction (MAE) from *Nannochloropsis* sp. microalgae were studied using a low cost and green solvent, namely brine (NaCl) solution. The kinetic modelling of the lipid extraction was performed to evaluate the mechanism of the lipid mass transfer using different extraction models, including Fick’s Law, First and Second-order Rate Law and the Patricelli mathematical model. The Patricelli mathematical model described the kinetics of lipid extraction well, with the highest average values of determination coefficient (R^2^ ≥ 0.952) and the lowest average values of mean relative percentage deviation (MRPD ≤ 8.666%). The lipid analysis indicated a positive influence of the microwave temperature and time on the quantity and quality of extracted lipids. SEM analysis of spent microalgae clearly shows an increase in the distorted cell with increase microwave temperature and time, which could be directly correlated to the mechanism of the MAE-brine technique.

## 1. Introduction

*Nannochloropsis* sp. microalgae are considered a promising microalga for the pharmaceutical industry due to its high lipid content with high levels of polyunsaturated fatty acids (PUFAs), especially omega-3 eicosapentaenoic acid (EPA) [1]. Commonly, there are several steps involved in microalgal lipid production, namely cultivation, harvesting, lipid extraction, separation and later, FA purification. Among these steps, the lipid extraction step is a crucial step for enhancing the quality and quantity of lipid production from microalgae [2]. The microwave-assisted extraction (MAE) technique has enormous potential as a promising method for microalgal lipid extraction, as it is non-intricate and eco-friendly [3], and useful for lipid extraction due to the high heating rate [3], minimal amount of solvent usage [4], lower operating cost and fast extraction time [5]. Moreover, utilizing a green and cheaper solvent such as brine can contribute to further reduction of the extraction cost, food safety and environmental pollution [6].

The recent extraction developments are mainly focused on the enhancement of product yield through the assistance of non-conventional techniques such as supercritical [7], ultrasonic-assisted [8], microwave-assisted [4] and electric field extrac tion [9]. The mass transfer parameters of lipid extraction from microalgae via conventional techniques have also been explained through thermodynamic and kinetic models [10]. Of the two types of modelling, the latter provides valuable information for scaling up the extraction process [10]. However, for the non-conventional techniques kinetic modelling has yet to be thoroughly studied, except for continuous solvent extraction using the supercritical method [11,12]. Only a few MAE [4] and UAE [13] extraction batch process kinetic modelling studies were attempted for primary products such as lipids. Others have modelled the extraction of bioactive products such as phenols via MAE [14] and alkaloids via UAE [15].

There are many kinetic models via mathematical approaches that have been used to model extraction kinetics from plants. The models can be either theoretically derived, such as from Fick’s law and chemical kinetic equations (Rate Law) or empirically formulated. The diffusivity in Fick’s Law is an important property indicating the rate of mass transfer and is very useful for equipment design [16]. A derivation of Fick’s Law extraction was used to model supercritical extraction of ginger rhizome [17], hexane extraction of ground sunflower seeds in a continuously stirred batch system [16] and the steam explosion of sumac fruit [18].

Another approach of modelling is using the chemical kinetic equation or Rate Law. Extraction models based on First and Second-order Rate Law were applied in conventional and non-conventional extractions, though the latter is mostly used. First-order kinetic modelling was used to describe the extraction of phenols from tea in MAE [14]. Meanwhile, Second-order kinetic modelling was used to explain the UAE of pomegranate peel [19], water extraction of ground pomegranate marc [20], and water and ethanol/water extraction of *Fumaria officinalis* L. [21].

Empirical equations have also been developed to explain the two distinct phases in the product extracted from plants. Osburn and Katz [22] suggested using the mass transfer modelling in solid particles derived from Fick’s Law to consider the two-step extraction in order to achieve better modelling. This mass transfer model might be the basis of the empirical model proposed by Patricelli [23]. These empirical models are believed to be more suitable in modelling non-conventional extraction processes such as MAE, UAE, and electric field-assisted extraction as they cannot be described accurately by the theoretical equations such as those derived from diffusion and chemical kinetics theories [10]. The most common empirical model used is the model developed by Patricelli [23] and then later used and further modified by So and MacDonald [24] for the hexane extraction of oil from rapeseed. Patricelli’s model has been widely employed to study oil extraction using MAE from olive cake [4], UAE from black seed [25] and pressure-assisted extraction of phenols from tea [26].

Therefore, the objective of this study was to develop a mathematical model that describes the kinetics of lipid extraction from *Nannochloropsis* sp. microalgae using the MAE-brine green technique, in which the kinetic data have been studied using different models. Moreover, the effects of MAE-brine temperature on the quality of extracted lipids were analyzed. 

## 2. Results and Discussion 

### 2.1. MAE-Brine Kinetics Modelling 

The kinetic experiments were conducted for 30 min using MAE-brine solvent under the previously obtained optimum extraction conditions [6]. Lipid extraction were done using 10% (*w*/*v*) NaCl as solvent and 5% (*w*/*v*) solid loading at extraction temperatures of 60, 70, 80, 90 and 100 °C. Table 1 lists the experimental results for the lipid extraction. The results show an increase in the lipid yield with increasing extraction time and this is more prominent at higher temperatures. A clear jump of lipid yield is worth noting for extraction at 100 °C (between 3 and 6 min), which is not observed at the lower temperatures studied. The highest extraction temperature and longest extraction time of 100 °C and 30 min resulted in the highest lipid yield of 16.1%. According to previous work [6], this yield was three-fold better than that of Soxhlet extraction and only 2% less than achieved with the Bligh and Dyer (B&D) lipid extraction method, which utilizes harmful solvents.

The kinetic approach modelling would allow a better understanding of the extraction rates and possible extraction mechanisms of the lipids. Four kinetic models, namely Fick’s law, First and Second-order Rate Laws, and the Patricelli empirical model were employed to fit the experimental kinetic data of lipid extraction from *Nannochloropsis* sp. microalgae. Table 2 presents the values of R^2^ and MRPD for the various kinetic models. Among these, Second-order Rate Laws and Patricelli empirical model fit the experimental data the best, showing the highest values of R^2^ and the lowest values of MRPD. Therefore, the kinetic parameters of these models at different extraction temperatures are determined, as presented in the following subsections. 

#### 2.1.1. Second-Order Rate Law

**Figure 1:**
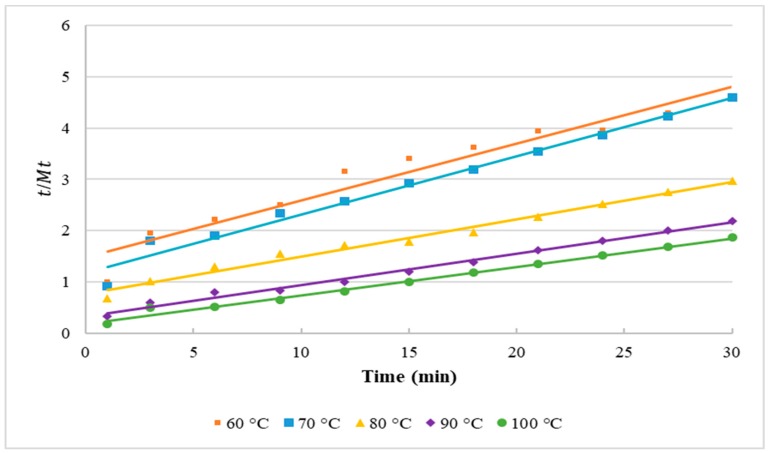
Second-order Rate Law kinetic model of *Nannochloropsis* sp. microalgal lipids using MAE-brine at different extraction temperatures.

The evaluation of the kinetic behaviour of extracted lipids from *Nannochloropsis* sp. microalgae using the MAE-brine method was conducted using Second-order Rate Law. For all studied temperatures, the plots of *t/M_t_* versus *t* resulted in a linear function, as shown in Figure 1. Table 3 presents the Second-order kinetic parameters, the Second-order Rate Law coefficients ($k_{2}$) and lipid yields at saturation $\left(M_{\infty }\right)$ The increase in the temperature results in an increment in $k_{2}$ values. The highest $k_{2}$ value of 0.016 was observed at the highest tested temperature of 100 °C, while the run at the lowest microwave temperature (60 °C) resulted in the lowest $k_{2}$ value of 0.008. A similar trend was reported when the second-order kinetic model was applied to the extraction of camptothecin from the plant *Nothapodytes nimmoniana* using microwave-assisted process intensification [15]. Moreover, the values of $M_{\infty }$ show a significant increase corresponding to extraction temperature, where the highest lipid yield of 18.08% was obtained at the highest tested temperature (100 °C). The values of $M_{\infty }$ indicate that the concentration of lipids in the extraction solvent eventually increases to a specific level.

The extraction mechanism of the second rate-order model means that the lipid extraction occurs through two simultaneous processes [27]. The amount of lipid extracted increased rapidly with time at the beginning of the extraction and then increased slowly with time until the end of the extraction process [21,28,29]. The kinetic results in Table 1 are in agreement with this hypothesis, where the lipid yield increased rapidly at the beginning, followed by a slow increase thereafter.

The extraction solvent penetrated the solid matrix by effective diffusion, then the lipid dissolved in the solvent until it is saturated. Next, the lipid saturated solvent diffused through the matrix surface by diffusion into the bulk solvent. The second-order rate mechanism takes place through three different steps. The first step is the equilibrium phase in which the solubilization phenomena and intervene partition occurs, where the substrate is taken away from the surface at an approximately constant velocity. Then, this step is followed by an intermediary transition phase. During this phase, the mass transfer resistance between the solid and liquid interface starts to appear. In this step, the mass transfer is theorized to be through convection and diffusion. In the last step, the solute overcomes the interactions with the solid matrix then diffuses into the extraction solvent. The rate of extraction in this step is low, characterised by the extraction of the solute through a diffusion mechanism [30]. Moreover, the increase of the solute concentration in the last step can explain the reason for the decrease in the rate of diffusion [27]. The study of Kusuma and Mahfud [31] explained the kinetic extraction of sandalwood oil using microwave-assisted hydrodistillation through second-order Rate Law. They claimed that the extraction took place in two stages: an intense dissolution at the beginning followed by a strong scrubbing of the soluble molecules. 

**Figure 2:**
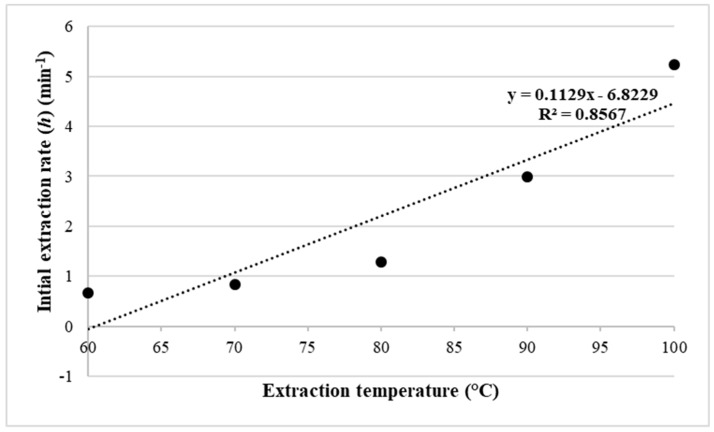
Effect of the extraction temperature on the initial extraction rate of *Nannochloropsis* sp. microalgae using MAE-brine according to the second-order Rate Law model.

The initial extraction yields (*h*) were calculated at each microwave temperature using Equation (17) (see Section 3). Figure 2 shows a positive correlation between *h* and temperature with a determination coefficient of 0.86. This correlation proves that the MAE technique can extract a high amount of lipid at a short time at a higher temperature. 

**Figure 3:**
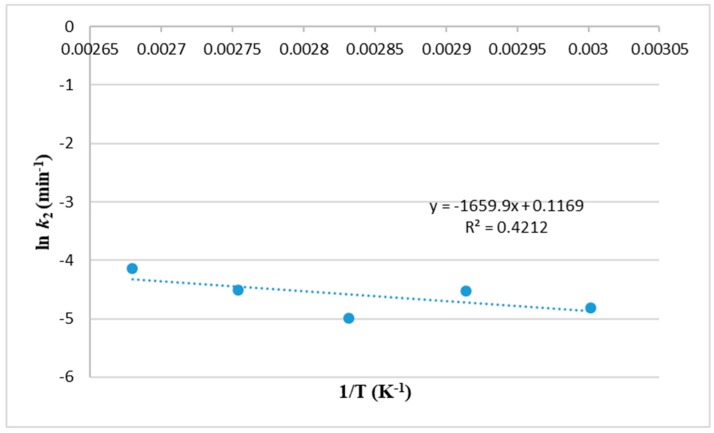
Relationship between temperature and the second-order extraction rate coefficient, ln *k*_2_, for extraction of lipids from *Nannochloropsis* sp. microalgae using MAE-brine.

The energy required for lipid extraction according to the Second-order model was calculated through plotting ln *k*_2_ versus *1/T* according to Equation (23) (see Section 3) and plotted in Figure 3. The value of activation energy ∆*E_a_*_2_ obtained with a low determination coefficient (R^2^ = 0.42) was 13.8 kJ/mol.

#### 2.1.2. Empirical Model—Patricelli Kinetic Model

**Figure 4:**
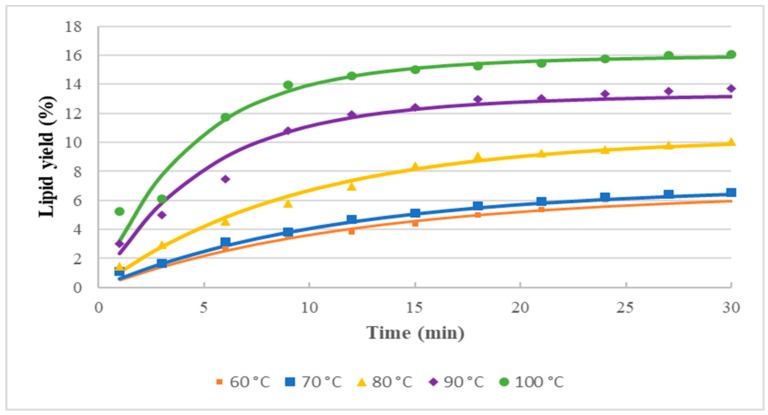
Relationship between time and extraction yield of *Nannochloropsis* sp. microalgal lipids using MAE-brine, according to Patricelli kinetic model.

The empirical kinetic model developed by Patricelli was examined to fit the lipid extraction yield. The experimental data were regressed using Polymath software to fit the Patricelli model Equation (18) (see Section 3). Figure 4 presents the profiles of experimental values and calculated values of the lipid yields at five different extraction temperatures via the MAE-brine technique. It can be observed that the extracted lipid is rapidly increased at the beginning of the MAE extraction process from up to 10 min for all temperatures then slowly increased thereafter. These two transition phases of rapid and slow lipid yield extraction were also reported previously by Patricelli [23] and deduced as to have two extraction mechanisms-rapid washing and slow diffusion, respectively. 

**Figure 5:**
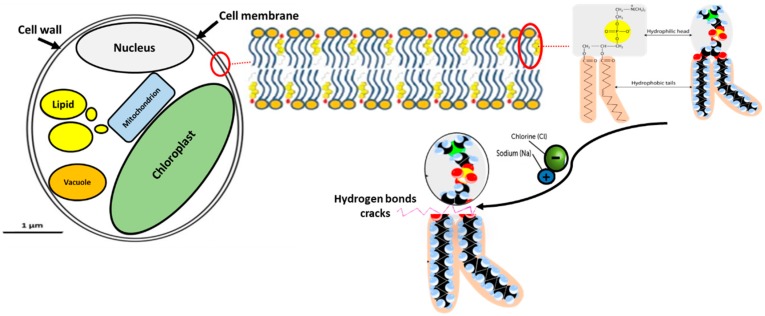
Illustration the effect of NaCl ions on the microalgal cell wall cracks.

In biological samples, the presence of Na^+^ Cl^−^ ions had an effect on the change of membrane electrostatics, thus influencing the lipid self-diffusion, in which the diffusion coefficient value increases [32,33]. Choi and his co-authors showed in their study that the hydrogen bonds in the microalgal cell membrane are affected by the presence of ions [34]. Even if the microalgal cells are not destroyed, the broken hydrogen bonds allow the content in the cell such as lipids and proteins to diffuse into the bulk solvent. Another possible explaination is given by the study of Pan et al. [35]. They related the enhancement of the lipid extraction from *C. sorokiniana*, *N. salina*, and *G. sulphuraria* species to the migration of ions within the altering electric field that was capable to disrupt microalgal cells. Therefore, the presence of Na^+^ and Cl^−^ ions in the extraction solvent resulted in the cracks that disrupt the microalgal cell wall, thereby resulted in an abrupt high increment of lipid yield (Figure 5). Additionally, the dielectric properties of the solvent brine are proportional to the temperature [36], hence localized heating occurs. This phenomenon may have decreased the viscosity of lipids thus facilitating the dissolution of the lipid into the bulk solvent [4]. 

The second phase, namely the slow diffusion phase, is characterized by a slower increment of the amount of lipids extracted. It is inferred that the remaining lipids from inside the ruptured cells and in the cell walls (those cells that did not rupture) diffuse slowly into the solvent.

Table 4 presents the maximum lipid yields during both phases and the total lipid yield, as well as the mass transfer coefficients for both phases. The washing mechanism for lipid extraction dominates over the diffusion mechanism in all of the temperatures studied. The mass transfer coefficients of the washing stage are 2 to 3 times greater than that of the diffusion stage. Additionally, the mass transfer coefficients for the washing phase increased with increasing temperature with the highest value of 0.245 min^−1^ at 100 °C and lowest at 60 °C (0.091 min^−1^). 

The mass transfer coefficients for the diffusion phase increased almost linearly with increasing temperature but it is less significant compared to the washing phase. These results confirm the effectiveness of MAE-brine at beginning of the extraction (washing phase), thus releasing more lipids during the washing phase. The enhancement of the mass transfer with increasing temperature during the diffusion phase may be due to the increase of kinetic energy of the lipid particles in the cracked microalgal cells. As the kinetic energy of the lipid particles increases, the lipids begin to move faster and more significantly [37].

Likewise, the maximum lipid yields for the washing phase are higher than that of the diffusion phase at all microwave temperatures studied by a factor of 3.5 to 6.2. This confirms that most of the *Nannochloropsis* sp. microalgal lipids were extracted during the washing phase through cell cracks induced by the microwave irradiation and brine solvent. It can be deduced that the enhancement on the microalgal lipids extraction is mainly dependent on the microwave temperature, in which the lipid yield values of the washing stage increased significantly with increasing temperature. Table 4 shows the calculated total lipid yield ($M_{\infty }$) at each temperature depending on the lipid yield values at equilibrium for washing and diffusion phases.

**Figure 6:**
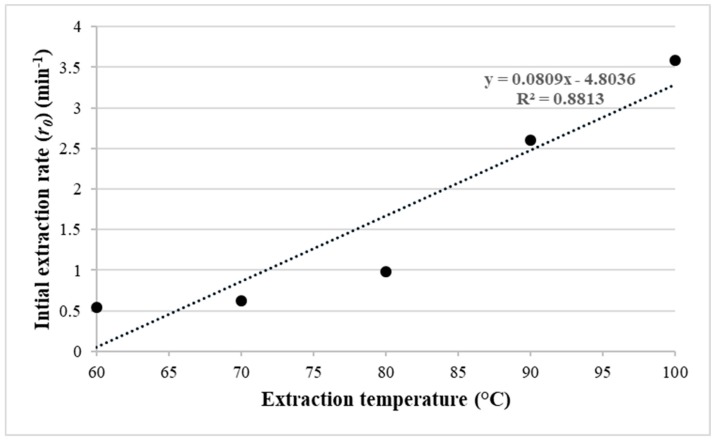
Effect of the extraction temperature on the initial extraction rate of *Nannochloropsis* sp. microalgae using MAE-brine, according to Patricelli kinetic model.

Figure 4 shows that the microalgal lipid yields increase quickly at the beginning of the extraction process as calculated using Equation (20) (see Section 3). To confirm this phenomenon, the calculations of the extraction rate (*r*), according to Equation (19) (see Section 3) are presented in Table 5. The calculation results show a significant increase in the extraction rate of the lipid yield at the beginning of the extraction process but quickly decreased subsequently for all temperatures. It is also noticed from Figure 6 that the initial rate (*r*_0_) at the very beginning of the extraction process increased with the extraction temperature. The initial rate at 100 °C extraction temperature is six times higher than that of 60 °C, which explains the higher MAE efficiency at a higher temperature.

**Figure 7:**
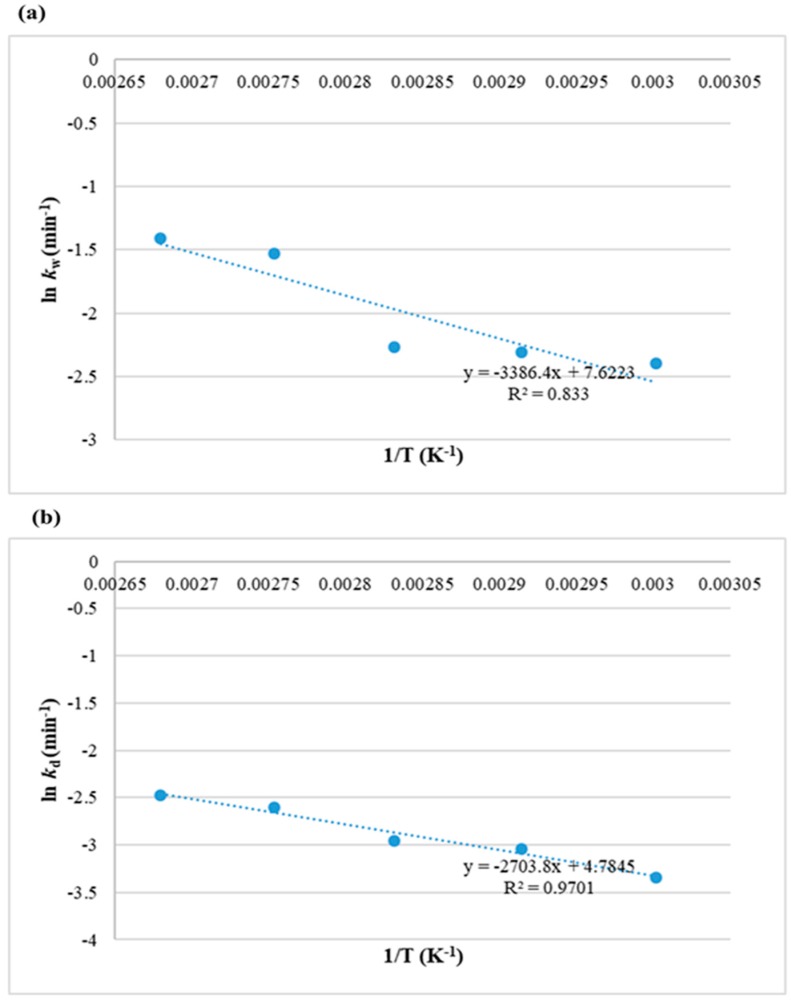
Relationship between temperature and Patricelli kinetic model coefficients at different phases (**a**) washing phase and (**b**) diffusion phase, for extraction of lipids from *Nannochloropsis* sp. microalgae using MAE-brine.

The effect of temperature on the mass transfer coefficient was determined using the Arrhenius equation for each washing and diffusion phase. Figure 7a,b show high coefficient of determination values of 0.83 and 0.97 obtained as a result of plotting $lnk_{w}$ and $lnk_{d}$ versus 1/*T*, respectively. The calculated activation energy value for the washing phase $\Delta E_{aw}$ was 28.1 kJ/mol, while it was lower for the diffusion phase ($\Delta E_{ad}$ = 22.4 kJ/mol). Patricelli kinetic model calculated that the minimum energy required to extract *Nannochloropsis* sp. microalgal lipids is 50.5 kJ/mol.

Among the kinetic models used, the empirical model Patricelli gives the most accurate fit with the highest determination coefficient (R^2^ > 0.95) and the lowest mean relative percentage deviation (MRPD < 8.66). The empirical kinetic models are more appropriate in describing the extraction process involved in assisted techniques such as microwave as they cannot be adequately described theoretically [10].

Schneeberger et al. [38] found that the extraction mechanism of oil extracted from hazelnut cannot be controlled by diffusion extraction only. Patricelli’s kinetic model has successfully explained the kinetics of oil extraction from olive cake [4], black seeds [25], and green tea [26] using non-conventional methods such as the microwave, ultrasound, and pressure-assisted extractions, respectively. 

### 2.2. Lipid Quality Results

**Figure 8:**
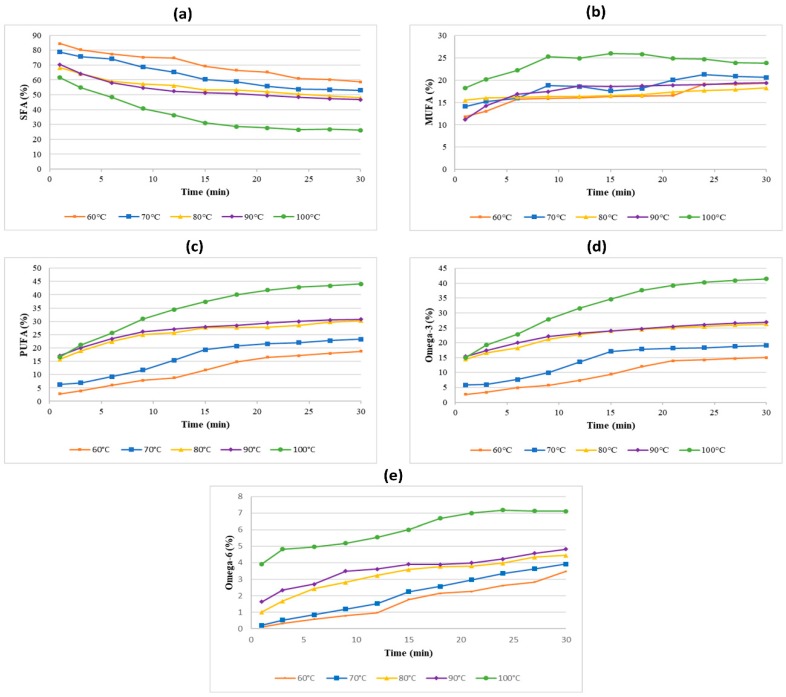
The percentages of (**a**) SFAs, (**b**) MUFAs, (**c**) PUFAs, (**d**) omega-3 fatty acids and (**e**) omega-6 fatty acids obtained from *Nannochloropsis* sp. microalgae using MAE-brine at different temperatures.

The fatty acid compositions were analyzed at each temperature used. Figure 8a shows the percentage of SFAs during the extraction process. The percentage of SFAs decreased with time for all temperatures and was more prominent for the higher temperatures. Although, the total amount of all FA types did not decrease when the PUFAs extraction rate increased, the percentage of other types of fatty acids such as SFAs is adversely affected. The percentage of MUFAs and PUFAs increased with extraction time, as illustrated in Figure 8b,c, respectively. The PUFA increment is more significant as compared to that of MUFAs. This finding is similar to previous work by Balasubramanian et al. [39], where they found that the SFAs percentage of *Scenedesmus obliquus* microalgae decreased with microwave extraction time, corresponding to an increase of the PUFA percentage.

According to Pereira et al. [40], PUFAs are the main fatty acids class found in the microalgal cell wall. The highest PUFAs percentage found at the higher temperature of MAE extraction (100 °C) could be because more cell walls were ruptured and cracked at this temperature. The proportional trend of the PUFAs percentage with temperature also supports this hypothesis. Additionally, at higher temperatures, the mass transfer rate is proven higher as per calculated in the subsection above. Another possible reason is that less degradation of microalgal thermolabile compounds the as shorter extraction time due to the high heating rate in MAE technique although the high temperature was used [2]. The study of Tyagi and Vasishtha [41] reported that PUFAs from soybean oil and vanaspati become saturated at high temperature and time by deep-frying at 190 °C, showing that heating treatment at a longer time increase saturation. It was reported that a low thermal degradation of carotenoids obtained from olive oil were detected when microwave heating was applied compared to the rapid degradation when conventional heating was used [42]. The highest PUFAs percentage at 44.1% (25.2 mg/g) was obtained at an extraction temperature of 100 °C at 30 min.

The profiles of essential omega-3 and omega-6 fatty acids at different extraction temperatures were also plotted during the 30 min microwave irradiation time, as illustrated in Figure 8d,e, respectively. The percentages of omega-3 and omega-6 increased with time for all tested temperatures. The highest temperature also resulted in the highest extraction rate and the yield is similar to that of PUFAs profile with a more significant increase of omega-3 percentage. In this study, the PUFAs analysis represents only seven of the 20 tested FAs, where omega-3 fatty acid is the main constituent of the total PUFAs. The study by Balasubramanian et al. [39] reported a similar deduction of the effect of microwave extraction time and temperature on the omega-3 and omega-6 percentages. Percentage of omega-3 and omega-6 from Scenedesmus obliquus microalgae also increased to around 50% at the higher extraction temperature and time. The highest omega-3 percentage at 41.4% (23.6 mg/g) was detected at an extraction temperature of 100 °C at 30 min for this study. The EPA fatty acid is the most abundant compound affecting the total omega-3 values, accounting for 31.5% (17.9 mg/g) of the total fatty acids.

### 2.3. Morphology of the Microalgae Cells 

**Figure 9:**
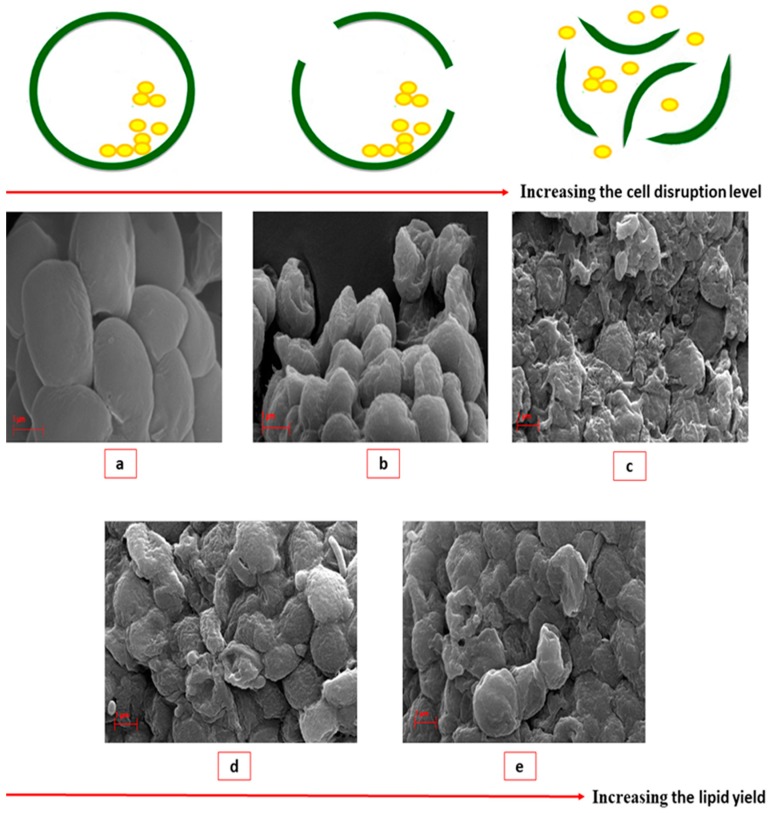
The picture of the effect of the cell cracks on the lipid recovery using SEM micrograph (10 k) for *Nannochloropsis* sp. microalgae (**a**) before MAE, (**b**) at MAE conditions of 100 °C and 3 min, (**c**) at at MAE conditions of 100 °C and 30 min, (**d**) at MAE conditions of 60 °C and 30 min and (**e**) at MAE conditions of 80 °C and 30 min.

The effect of the MAE-brine technique on the cell morphology is supported by the images of microalgal cells captured by scanning electron microscopy (SEM). Figure 9a–e show the effects of extraction temperature and time on cell integrity. Figure 9a confirms the ovoidal shape of the *Nannochloropsis* sp. cells. The SEM images in Figure 9b,c demonstrate a more cluster of distorted biomass with the increase of both extraction temperature and time in comparison to the original cells appearing as totally distorted cells at 30 min. Figure 9c–e clearly show an increase in the wrinkles and cracks on the microalgal surface as a result of increasing temperature at 100, 60 and 80 °C, respectively. These results support the enhancement of lipid extraction yield at optimum extraction conditions using the MAE technique as a result of microalgal cells cracks. The SEM results are in agreement with the previous work of Juin et al. [43], where they found a major changes in the cell morphology of freeze-dried *Porphyridium purpureum* when the MAE temperature was increased.

## 3. Materials and Methods

### 3.1. Materials

Dried powdered *Nannochloropsis* sp. microalgae (class of *Eustigmatophyte*) were purchased from Xi’an Lyphar Biotech Co., Ltd. (Xi’an, Shanxi, China). The microalgal cells were cultivated using nitrate at a concentration of 1.6 g∙L^−1^ and phosphate at a concentration of 0.2 g∙L^−1^ NaH_2_PO_4_ at pH 8, before being freeze-dried and vacuum-packed for further use. The chemicals used, namely hexane (C_6_H_14_), chloroform (CHCl_3_), methanol (CH_3_OH), acetone (C_3_H_6_O) and sodium chloride (NaCl) were procured from R&M Chemicals (London, UK), and of analytical grade, while hydrochloric acid (HCl) was purchased from Sigma-Aldrich Co. (Saint Louis, MO, USA).

### 3.2. Microwave-Assisted Extraction (MAE)

#### 3.2.1. Microwave Extractor Setup

**Figure 10:**
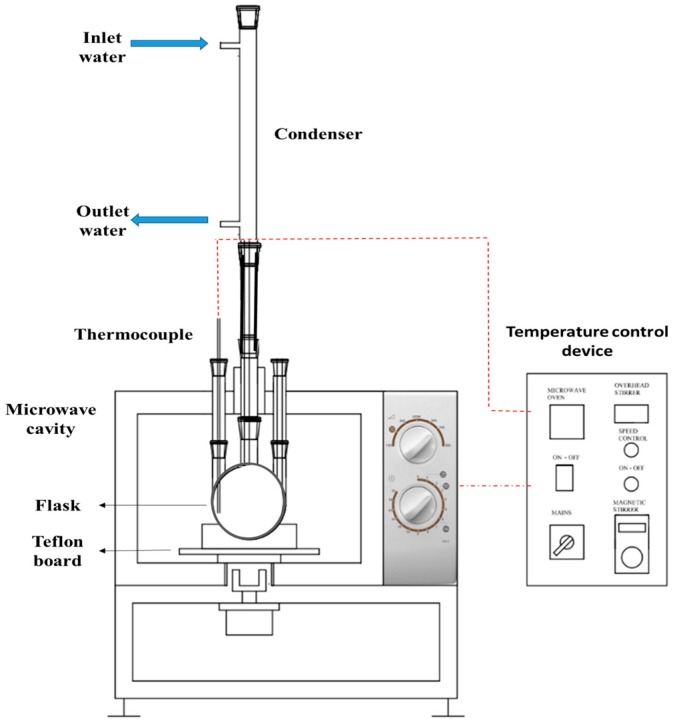
Schematic of the MAE system.

In this experiment, a modified domestic microwave with a frequency of 2.45 GHz (ME711K, Samsung, Port Klang, Selangor, Malaysia) combined with a temperature control device was used, in which the microwave has a maximum power of 800 Watt. The temperature controlling device can control temperature to a maximum of 240 °C. At the top of the microwave cavity, there are two 25 mm round holes, in which the centre hole connects the round bottom flask (which contains the algal sample) with a condenser, while the side hole facilitates the thermocouple instalment in the extraction solvent. The holes are sealed with custom-made Teflon caps to avoid the leak of microwave radiation. Figure 10 presents a schematic drawing of the microwave system.

#### 3.2.2. Experimental Procedures

The microwave extraction was performed in a 250 mL round bottom borosilicate flask. For the kinetic experiments, the optimised conditions from a previous work [6] were used with salt concentration of 10% (*w*/*v*) and solids loading of 5% (*w*/*v*). The runs were set for a maximum time of 30 min. The kinetic study experiments were performed at temperatures of 60 to 100 °C at 10 °C intervals. All sets of experiments were run in triplicates.

A few millilitres (~5 mL) of the hexane solvent was added to the solvent and biomass to ensure full lipid recovery after each MAE treatment. Then, the mixture was centrifuged (3500 rpm, 5 min) and the upper layer containing lipid dissolved hexane was collected. After drying the hexane solvent, the lipid fraction was recovered and determined gravimetrically. The yield was calculated according to Equation (1):

(1) $$\mathrm{Lipid}\;\mathrm{yield}\;(\%)=\frac{\mathrm{mass}\;\mathrm{of}\;\mathrm{extracted}\;\mathrm{lipid}\;\left(g\right)}{\mathrm{mass}\;\mathrm{of}\;\mathrm{dried}\;\mathrm{microalgae}\;\left(g\right)}\times 100\%$$

#### 3.2.3. Transesterification of Lipid

The transesterification reaction following the method described by Lewis et al. [44] was used to produce fatty acid methyl esters (FAMEs) from the extracted *Nannochloropsis* sp. microalgal lipids. First, 3 mL of CH_3_OH/HCl/CHCl_3_ (10:1:1 *v*/*v*/*v*) mixture was added to the extracted lipids and vortexed for 15 s. Then, the sample mixture was heated for an hour at 90 °C. Once the mixture cooled, 1 mL of distilled water was added. Next 2–3 mL C_6_H_12_/CHCl_3_ (4:1 *v*/*v*) mixture was added to form two layers. The upper layer, which contained FAME fraction, was collected, filtered, and evaporated to determine FAME yield according to Equation (2). For further GC analysis, the fraction of FAMEs was dissolved in 1.5 mL hexane.

(2) $$\mathrm{FAMEs}\;\mathrm{yield}\;(\mathrm{mg}/g)=\frac{\mathrm{mass}\;\mathrm{of}\;\mathrm{extracted}\;\mathrm{FAMEs}\;\left(\mathrm{mg}\right)}{\mathrm{mass}\;\mathrm{of}\;\mathrm{dried}\;\mathrm{microalgae}\;\left(g\right)}$$

#### 3.2.4. FAME Analysis Using GC-FID

FAMEs compositions were determined using gas chromatography (Agilent 6890 GC, Saint Paul, MN, USA) equipped with a flame ionization detector (GC/FID). A polar ZB-WAX column (Zebron, Newport, CA, USA) with a 30 m length, 0.25 mm diameter and 0.25 μm film thickness was used. Hydrogen at 3 mL/min was used as the gas carrier. The FAMEs fraction sample was injected at a temperature of 250 °C, and it was operated in a splitless mode. After 1 min of the initial oven temperature of 100 °C, the temperature was ramped up to 230 °C at a rate of 5 °C/min. The temperature for detector was fixed at 260 °C. The quantities of FAMEs in each sample were determined based on the standard of FAME mixture for 20 fatty acids provided by Marine Oil FAME Mix (Restek, Bellfonte, PA, USA).

### 3.3. Kinetic Models

The extraction kinetics of *Nannochloropsis* sp. microalgal lipids using MAE-brine technique at different microwave temperatures of 60, 70, 80, 90, and 100 °C were studied. Four kinetic models, namely Fick’s Law, First and Second-order Rate Law, and empirical model (Patricelli model) were used to model the lipid extraction.

#### 3.3.1. Fick’s Law

A modified Fick’s Law model for spherical geometry particle diffusion in the non-stationary state was used. According to Perez and Crapiste [16], the modelling of Fick’s Law follows Equation (3) below:

(3) $$\frac{M_{t}}{M_{\infty }}=1-\overset{\infty }{\underset{n=1}{\sum }}A_{n}exp\left[-B_{n}t\right]$$

where $M_{t}$ and $M_{\infty }$ are the amount of lipid that diffused at time t and infinite time, respectively. $A_{n}$ is the model constant and $B_{n}$ is the diffusion rate constant which was fitted to the experimental data. The model takes into account the initial time at which the rapid phase (non-diffusive) takes place. During the rapid phase, the solute (microalgal lipids) released due to cell rupture by the treatment process such as MAE is considered as $M_{o}$ Therefore, these conditions and the boundary conditions to solve Equation (3) are the following: at *t* = 0, there is not solute in the solvent. Which is the initial stage (washing stage), M is equal to $M_{o}$ At any time *t*, M is equal to $M_{t}$ and at infinite time, *t*, M = $M_{\infty }$ The equation can now be solved to obtain Equation (4), after rearrangement:

(4) $$\frac{M_{t}}{M_{\infty }}=1-\left(1-\frac{M_{t}}{M_{\infty }}\right)\overset{\infty }{\underset{n=1}{\sum }}A_{n}exp\left[-B_{n}\left(t-t_{o}\right)\right]$$

After sufficiently long times, nearing to equilibrium, Equation (4) can be simplified into:

(5) $$\frac{M_{t}}{M_{\infty }}=1-A\;exp\left[-B_{1}t\right]$$

where the pre-exponential coefficient *A* is given by:

(6) $$A=\left(1-\frac{M_{t}}{M_{\infty }}\right)A_{1}\;exp\left[B_{1}t\right]$$

and where the coefficients $A_{1}$ and $B_{1}$ for spherical geometry can be expressed as:

(7) $$A_{1}=\frac{6}{\pi^{2}}$$

(8) $$B_{1}=\frac{D_{e}\pi^{2}}{R^{2}}$$

The modelling of Fick’s Law follows the Equation (5), which was used to calculate the lipid yield for 30 min of extraction duration. Rearrangement of the equation yields:

(9) $$\frac{M_{\infty }-M_{t}}{M_{\infty }}=A\;exp\left[-B_{1}t\right]$$

Both sides were subjected to natural log expansion to linearize the above equation and was solved as:

(10) $$ln\left(\frac{M_{\infty }-M_{t}}{M_{\infty }}\right)=ln\;A+-B_{1}t$$

The plot of $ln\left(\frac{M_{\infty }-M_{t}}{M_{\infty }}\right)$ versus *t* yielded the slope and intercept of a linear equation. The slope is equal to $-B_{1}$ and the intercept is ln A, which was solved to obtain $B_{1}$ and A. The coefficient $B_{1}$ was used to calculate the effective diffusion coefficients, $D_{e}$ using Equation (8).

#### 3.3.2. First-Order Rate Law

The rate of products extracted into the solvent via First-order Rate Law is given in Equation (11).

(11) $$\frac{dM}{dt}=k_{1}M$$

where $k_{1}$ is the First-order rate constant. Integrating Rate Law with boundary conditions at which *t* = 0, M is equal to $M_{o}$ and at any time t, M is equal to $M_{t},$ Equation (11) becomes

(12) $$M_{t}=M_{o}exp\;\left(k_{1}t\right)$$

The equation is linearized to solve for First-order Rate constant $k_{1}$ and $M_{o}$.

(13) $$lnM_{t}=lnM_{o}+k_{1}t$$

The plot of $ln\left(M_{t}\right)$ versus t yielded the slope and intercept of a linear equation. The slope is equal to first-order Rate constant $k_{1}$ and the intercept is $ln\;M_{o}.$

#### 3.3.3. Second-Order Rate Law

Kinetic model for the extraction of products from plants has also been adequately explained by Second-order Rate Law [15,21,28]. The dissolution rate of the product in the cell-matrix can be given by:

(14) $$\frac{dM}{dt}=k_{2}{\left(M_{\infty }-M\right)}^{2}$$

where M and $M_{\infty }$ are the amounts of extracted lipids at any time and saturation, respectively. $k_{2}$ is the Second-order extraction rate constant. By considering the initial and boundary conditions where at *t* = 0, M is zero and at any time t, M equals to $M_{t}$, and integrating the Second-order Rate Law, Equation (14) becomes:

(15) $$M_{t}=\frac{M_{\infty }^{2}k_{2}t}{1+M_{\infty }k_{2}t}$$

Further transformation of Equation (15) to a linear form, the extraction rate can be written as Equation (16) below:

(16) $$\frac{t}{M_{t}}=\frac{1}{k_{2}M_{\infty }^{2}}+\frac{t}{M_{\infty }}$$

A plot of $\frac{t}{M_{t}}$ versus *t* yielded the slope and intercept of a linear equation. The slope is $\frac{1}{M_{\infty }}$ and the intercept is $\frac{1}{k_{2}M_{\infty }^{2}}$ where the Second-order Rate constant $k_{2}$ can be calculated. Initial extraction rate, h was calculated using Equation (17) below:

(17) $$h=k_{2}M_{\infty }^{2}$$

#### 3.3.4. Patricelli Empirical Model

The empirical model proposed by Patricelli [23] was used to explain the kinetic of the lipid extraction from solids is based on two simultaneous mechanisms as follows:

- Washing: the lipids are rapidly extracted through washing with the extraction solvent.
- Diffusion: the remaining lipids are slowly removed through diffusion to the extraction solvent.

Patricelli’s mathematical model is expressed in Equation (18):

(18) $$M_{t}=M_{w}\;\left(1-exp\;(-k_{w}t\right))+M_{d}\left(1-exp\;\left(-k_{d}t\right)\right)$$

where $M_{t}$ is the lipid yield at any time, $M_{w}$ is the lipid yield at equilibrium for the washing step, $M_{d}$ is the lipid yield at equilibrium for the diffusion step, $k_{w}$ is the mass transfer coefficient for the washing step, $k_{d}$ is the mass transfer coefficient for the diffusion step, and t is the time. The values of lipid yields at equilibrium ($M_{w}$ and $M_{d}$) and mass transfer coefficients ($k_{w}$ and $k_{d}$) were calculated using Polymath Software ver. 6.10 (CACHE Corporation, Austin, TX, USA).

The extraction rate for each kinetic point at different extraction temperature was determined from the first derivative of the Patricelli Equation (18) yielding Equation (19):

(19) $$r=\frac{dM}{dt}=M_{w}k_{w}exp\;\left(-k_{w}t\right)+M_{d}k_{d}exp\left(-k_{d}t\right)$$

At time 0, the initial rate ($r_{0}$), is given by:

(20) $$r_{0}={\left(\frac{dM}{dt}\right)}_{t=0}=M_{w}\;k_{w}+M_{d}\;k_{d}$$

### 3.4. Statistical Analysis

The validation of each model for lipid extraction was evaluated by measuring the variations between experimental and predicted values through the determination coefficient value, R^2^. Additionally, the goodness of fit for the kinetic models was estimated through a mean relative percentage deviation (MRPD), which is defined as in Equation (21):

(21) $$\mathrm{MRPD}=\frac{100}{N}\times \sum \frac{Me-M_{p}}{M_{p}}$$

where $M_{e}$ and $M_{p}$ are the experimental and predicted lipid yield, respectively, and *N* is the number of experimental runs. The kinetic models are considered to be accepted for the extraction process if R^2^ > 0.7 and MRPD < 10% [13,45].

### 3.5. Activation Energy

The variation of each model kinetic constant with temperature was calculated based on the Arrhenius equation defined by Equation (22):

(22) $$k=k_{0}\;exp\left[-\frac{\Delta E_{a}}{RT}\right]$$

where k is the model constant, $k_{0}$ is the temperature-independent factor, $\Delta E_{a}$ is the activation energy, R is the gas universal constant, and T is the absolute temperature. The transformation of this equation allows obtaining a linear relationship between each model constant and the reverse of temperature as presented in Equation (23); thus, the activation energy can be calculated:

(23) $$lnk=lnk_{0}-\frac{\Delta E_{a}}{RT}$$

### 3.6. Scanning Electron Microscopy

The *Nannochloropsis* sp. microalgae surface structure were analyzed using the Scanning Electron Microscopy (SEM) method, where the microalgae were examined before and after extraction with MAE-brine at different time and temperatures. Firstly, the microalgae sample was stabilized/fixed for 2 h with 3% glutaraldehyde buffered with a buffer of 0.1 M phosphate. Then, the mixture was washed for serval times with 0.1 M phosphate buffer. For the next 2 h, it was immersed in 1–2% osmium tetroxide in buffer solution (pH 7.2). After that and before being dried with CO_2_ at critical point using a critical point dryer (S00111087, BAL-TEC, Los Angeles, CA, USA), the mixture was dehydrated in acetone. The dried samples were then coated with gold in the Edwards vacuum system (BAL-TEC Bai-Tec Scd 005) after they were mounted on SEM stubs. Finally, the coated samples were ready to be imaged using the SEM instrument (S-34OON, Hitachi, Chiyoda-ku, Tokyo, Japan).

## 4. Conclusions

Kinetics modelling of lipid extraction from *Nannochloropsis* sp. microalgae using the MAE-brine technique was performed. To find the best fit that explains the extraction kinetics, different models, including Fick’s Law, First and Second-order Rate Laws, and Patricelli mathematical models were evaluated. Among these, the Patricelli mathematical model fits the best with the experimental results. In which, the statistical results show the highest average values of R^2^ (0.955–0.987) and the lowest average values of MRPD (6.749–8.666). According to the experimental data, the mathematical model coefficients were calculated. The higher washing transfer coefficients (*k_w_*) over diffusion coefficients (*k_d_*) indicates the efficiency of the proposed MAE-brine method during the washing stage. Moreover, the results show a significant impact of the extraction temperature and time on the lipid yield and PUFAs percentage as well as the omega-3 amount. The SEM results emphasized the changes in the microalgal cell morphology as a result of raising microwave extraction temperature and time.

## Figures and Tables

**Table 1:** The yield of microalgal lipids by MAE of *Nannochloropsis* sp. using MAE-brine at different extraction temperatures.

Time (min)	Lipid Yield (%)				
	60 °C	70 °C	80 °C	90 °C	100 °C
1	1.00 ± 0.11	1.08 ± 0.09	1.45 ± 0.04	2.99 ± 0.11	5.26 ± 0.12
3	1.53 ± 0.02	1.67 ± 0.01	2.94 ± 0.08	5.02 ± 0.03	6.09 ± 0.21
6	2.71 ± 0.15	3.15 ± 0.13	4.58 ± 0.01	7.47 ± 0.05	11.80 ± 0.03
9	3.59 ± 0.02	3.85 ± 0.03	5.78 ± 0.02	10.78 ± 0.08	14.00 ± 0.03
12	3.81 ± 0.04	4.66 ± 0.03	6.99 ± 0.10	11.93 ± 0.01	14.60 ± 0.11
15	4.39 ± 0.09	5.13 ± 0.05	8.39 ± 0.04	12.40 ± 0.01	15.00 ± 0.04
18	4.97 ± 0.03	5.64 ± 0.05	9.09 ± 0.03	12.97 ± 0.03	15.30 ± 0.10
21	5.34 ± 0.01	5.93 ± 0.10	9.25 ± 0.10	13.01 ± 0.01	15.40 ± 0.07
24	6.07 ± 0.02	6.22 ± 0.04	9.51 ± 0.11	13.35 ± 0.01	15.70 ± 0.11
27	6.29 ± 0.01	6.39 ± 0.12	9.82 ± 0.04	13.50 ± 0.02	16.00 ± 0.05
30	6.48 ± 0.01	6.52 ± 0.02	10.07 ± 0.05	13.73 ± 0.01	16.10 ± 0.02

**Table 2:** Statistical parameters of the kinetic modelling for MAE-brine of lipids from *Nannochloropsis* sp. microalgae.

Extraction Temperature (°C)	Kinetic Model							
	Fick’s Law		First-Order Rate Law		Second-Order Rate Law		Patricelli Empirical Model	
	R^2^	MRPD	R^2^	MRPD	R^2^	MRPD	R^2^	MRPD
60	0.977	10.645	0.828	21.037	0.933	9.942	0.977	8.666
70	0.927	17.759	0.764	25.442	0.971	6.297	0.987	6.749
80	0.927	18.058	0.747	26.615	0.975	6.284	0.967	7.729
90	0.838	24.045	0.670	24.071	0.985	7.888	0.977	6.767
100	0.788	24.109	0.619	21.248	0.991	6.786	0.955	6.926

**Table 3:** The extraction coefficient, yield at saturation, and statistical parameters for lipid extraction from *Nannochloropsis* sp. microalgae using MAE-brine according to the second-order Rate Law model.

Extraction Temperature °C	Second-Order Rate Law Coefficient (min^−1^)	Yield At Saturation (%)
	*k*_2_	$M_{\infty }$
60	0.008	9.06
70	0.010	8.82
80	0.010	13.75
90	0.011	16.45
100	0.016	18.08

**Table 4:** The extraction coefficients, yields at equilibrium, and statistical parameters for lipid extraction from *Nannochloropsis* sp. microalgae using MAE-brine, according to Patricelli kinetic model.

Extraction Temperature (°C)	Mass Transfer Coefficients (min^−1^)		Yield (%)		
	*k_w_*	*k_d_*	*M_w_*	*M_d_*	$M_{\infty }$
60	0.091	0.035	5.33	1.50	6.83
70	0.099	0.047	5.47	1.61	7.08
80	0.103	0.051	8.47	2.01	9.48
90	0.216	0.074	11.38	2.01	13.39
100	0.245	0.084	13.93	2.25	16.18

**Table 5:** The variation in the extraction rate of *Nannochloropsis* sp. microalgal lipid according to time at different MAE temperature.

Time (min)	Extraction Rate (min^−1^)				
	60 °C	70 °C	80 °C	90 °C	100 °C
0	0.54	0.62	0.98	2.60	3.59
1	0.49	0.56	0.88	2.11	2.84
3	0.42	0.47	0.73	1.40	1.78
6	0.32	0.36	0.55	0.76	0.89
9	0.25	0.27	0.41	0.43	0.46
12	0.19	0.21	0.31	0.24	0.25
15	0.16	0.16	0.23	0.14	0.14
18	0.12	0.12	0.18	0.09	0.08

## References

[1] Sukenik A., Zmora O., Carmeli Y. (1993). Biochemical Quality of Marine Unicellular Algae with Special Emphasis on Lipid Composition. II. *Nannochloropsis* sp.. Aquaculture.

[2] Lee J.Y., Yoo C., Jun S.Y., Ahn C.Y., Oh H.M. (2010). Comparison of Several Methods for Effective Lipid Extraction from Microalgae. Bioresour. Technol..

[3] Dvoretsky D., Dvoretsky S., Temnov M., Akulinin E., Peshkova E. (2016). Enhanced Lipid Extraction from Microalgae *Chlorella Vulgaris* Biomass: Experiments, Modelling, Optimization. Chem. Eng. Trans..

[4] Amarni F., Kadi H. (2010). Kinetics Study of Microwave-Assisted Solvent Extraction of Oil from Olive Cake Using Hexane: Comparison with the Conventional Extraction. Innov. Food Sci. Emerg..

[5] Refaat A., El Sheltawy S., Sadek K. (2008). Optimum Reaction Time; Performance and Exhaust Emissions of Biodiesel Produced by Microwave Irradiation. Int. J. Environ. Sci. Technol..

[6] Zghaibi N., Omar R., Kamal M., Mazlina S., Biak A., Radiah D., Harun R. (2019). Microwave-Assisted Brine Extraction for Enhancement of the Quantity and Quality of Lipid Production from Microalgae *Nannochloropsis* sp.. Molecules.

[7] Crossley J., Aguilera J.M. (2001). Modeling the Effect of Microstructure on Food Extraction. J. Food Process Eng..

[8] Cárcel J.A., García-Pérez J.V., Mulet A., Rodríguez L., Riera E. (2010). Ultrasonically Assisted Antioxidant Extraction from Grape Stalks and Olive Leaves. Physics Procedia.

[9] Boussetta N., Vorobiev E., Deloison V., Pochez F., Falcimaigne-Cordin A., Lanoisellé J.L. (2011). Valorisation of Grape Pomace by the Extraction of Phenolic Antioxidants: Application of High Voltage Electrical Discharges. Food Chem..

[10] Chan C.-H., Yusoff R., Ngoh G.-C. (2014). Modeling and Kinetics Study of Conventional and Assisted Batch Solvent Extraction. Chem. Eng. Res. Des..

[11] Díaz M.S., Brignole E.A. (2009). Modeling and Optimization of Supercritical Fluid Processes. J. Supercrit. Fluid.

[12] Oliveira E.L., Silvestre A.J., Silva C.M. (2011). Review of Kinetic Models for Supercritical Fluid Extraction. Chem. Eng. Res. Des..

[13] Fuad F.M., Karim K.A. (2017). Kinetics Study of Oil Extraction from *Calophyllum inophyllum* Seeds Using Ultrasonic-Assisted Extraction Technique. J. Phys. Sci..

[14] Spigno G., De Faveri D. (2009). Microwave-Assisted Extraction of Tea Phenols: A Phenomenological Study. J. Food Eng..

[15] Patil D.M., Akamanchi K.G. (2017). Ultrasound-Assisted Rapid Extraction and Kinetic Modelling of Influential Factors: Extraction of Camptothecin from *Nothapodytes nimmoniana* Plant. Ultrason. Sonochem..

[16] Perez E.E., Carelli A.A., Crapiste G.H. (2011). Temperature-Dependent Diffusion Coefficient of Oil from Different Sunflower Seeds During Extraction with Hexane. J. Food Eng..

[17] Kandiah M., Spiro M. (1990). Extraction of Ginger Rhizome: Kinetic Studies with Supercritical Carbon Dioxide. Int. J. Environ. Sci. Technol..

[18] Chen G., Chen H. (2011). Extraction and Deglycosylation of Flavonoids from Sumac Fruits Using Steam Explosion. Food Chem..

[19] Pan Z., Qu W., Ma H., Atungulu G.G., McHugh T.H. (2012). Continuous and Pulsed Ultrasound-Assisted Extractions of Antioxidants from Pomegranate Peel. Ultrason. Sonochem..

[20] Qu W., Pan Z., Ma H. (2010). Extraction Modeling and Activities of Antioxidants from Pomegranate Marc. J. Food Eng..

[21] Rakotondramasy-Rabesiaka L., Havet J.-L., Porte C., Fauduet H. (2007). Solid–Liquid Extraction of Protopine from *Fumaria officinalis*, L.—Analysis Determination, Kinetic Reaction and Model Building. Sep. Purif. Technol..

[22] Osburn J.O., Katz D.L.V. (1944). Structure as a Variable in the Application of Diffusion Theory to Extraction.

[23] Patricelli A., Assogna A., Casalaina A., Emmi E., Sodini G. (1979). Fattori Che Influenzano L’estrazione Dei Lipidi Da Semi Decorticati di Girasole. Riv. Ital. Sostanze Grasse.

[24] George C.S., Douglas G.M. (1986). Kinetics of Oil Extraction from Canola (rapeseed). Can. J. Chem. Eng..

[25] Abdullah M., Koc A.B. (2013). Kinetics of Ultrasound-Assisted Oil Extraction from Black Seed (*Nigella sativa*). J. Food Process. Preserv..

[26] Xi J., He L., Yan L. (2015). Kinetic Modeling of Pressure-Assisted Solvent Extraction of Polyphenols from Green Tea in Comparison with the Conventional Extraction. Food Chem..

[27] Kusuma H.S., Amelia P.D., Admiralia C., Mahfud M. (2016). Kinetics Study of Oil Extraction from Citrus Auranticum, L. by Solvent-Free Microwave Extraction. Commun. Sci. Technol..

[28] Ho Y.-S., Harouna-Oumarou H.A., Fauduet H., Porte C. (2005). Kinetics and Model Building of Leaching of Water-Soluble Compounds of Tilia Sapwood. Sep. Purif. Technol..

[29] Uhm J.T., Yoon W.B. (2011). Effects of High-Pressure Process on Kinetics of Leaching Oil from Soybean Powder Using Hexane in Batch Systems. J. Food Sci..

[30] Chemat F., Cravotto C. (2013). Microwave-Assisted Extraction for Bioactive Compounds.

[31] Kusuma H., Mahfud M. (2017). Comparison of Kinetic Models of Oil Extraction from Sandalwood by Microwave-Assisted Hydrodistillation. Int. Food Res. J..

[32] Rainer A.B., Agnieszka H., Thomas H., Helmut G. (2003). Effect of Sodium Chloride on a Lipid Bilayer. Biophys. J..

[33] Mobeen R., Elisabeth V. (2009). Effects of Sodium Chloride on Membrane Fusion and on the Formation of Aggregates of Potassium Channel KcsA in Membrane. Biophys. Chem..

[34] Choi S.-A., Oh Y.-K., Jeong M.-J., Seung W.K., Lee J.-S., Park J.-Y. (2014). Effects of Ionic Liquid Mixtures on Lipid Extraction from Chlorella Vulgaris. Renew. Energ..

[35] Pan J., Muppaneni T., Sun Y., Reddy H.K., Fu J., Lu X., Deng S. (2016). Microwave-Assisted Extraction of Lipids from Microalgae Using an Ionic Liquid Solvent [BMIM][HSO_4_]. Fuel.

[36] Sidell B.D., Hazel J.R. (1987). Temperature Affects the Diffusion of Small Molecules Through Cytosol of Fish Muscle. J. Exp. Biol..

[37] Anwar J., Shafique U., Rehman R., Salman M., Dar A., Anzano J.M., Ashraf U., Ashraf S. (2015). Microwave Chemistry: Effect of Ions on Dielectric Heating in Microwave Ovens. Arab. J. Chem..

[38] Schneeberger R., Villarroez M., Drapela N., Caire F., Castillo M. (1988). Cinética de Extracción de Aceite de Avellana. Grasas y Aceites.

[39] Balasubramanian S., Allen J.D., Kanitkar A., Boldor D. (2011). Oil Extraction from *Scenedesmus obliquus* Using a Continuous Microwave System—Design, Optimization, and Quality Characterization. Bioresour. Technol..

[40] Pereira H., Barreira L., Figueiredo F., Custódio L., Vizetto-Duarte C., Polo C., Rešek E., Engelen A., Varela J. (2012). Polyunsaturated Fatty Acids of Marine Macroalgae: Potential for Nutritional and Pharmaceutical Applications. Mar. Drugs.

[41] Tyagi V., Vasishtha A. (1996). Changes in the Characteristics and Composition of Oils during Deep-Fat Frying. J. Am. Oil Chem. Soc..

[42] El-Abassy R.M., Donfack P., Materny A. (2010). Assessment of Conventional and Microwave Heating Induced Degradation of Carotenoids in Olive Oil by VIS Raman Spectroscopy and Classical Methods. Food Res. Int..

[43] Camille J., Jean-René C., Valérie T., Anne-Laure G., Jean-Baptiste B., Nicolas J., Raymond K., Jean-Paul C., Laurent P. (2015). Microwave-Assisted Extraction of Phycobiliproteins from *Porphyridium purpureum*. Appl. Biochem. Biotechnol..

[44] Lewis T., Nichols P.D., McMeekin T.A. (2000). Evaluation of Extraction Methods for Recovery of Fatty Acids from Lipid-Producing Microheterotrophs. J. Microbiol. Methods.

[45] Moore D.S., Notz W., Fligner M.A. (2013). The Basic Practice of Statistics.

